# Advocates, interest groups and Australian news coverage of alcohol advertising restrictions: content and framing analysis

**DOI:** 10.1186/1471-2458-12-727

**Published:** 2012-08-31

**Authors:** Andrea S Fogarty, Simon Chapman

**Affiliations:** 1 Sydney School of Public Health, The University of Sydney, Edward Ford Building (A27), Sydney, NSW, 2006, Australia

**Keywords:** Alcohol policy, Content analysis, News reportage, Advertising restrictions

## Abstract

**Background:**

Legislating restrictions on alcohol advertising is a cost-effective measure to reduce consumption of alcohol. Yet Australia relies upon industry self-regulation through voluntary codes of practice regarding the content, timing and placement of alcohol advertising. Ending industry self-regulation was recommended by the National Preventative Health Taskforce; a suggestion contested by the drinks industry. Debates about emerging alcohol-control policies regularly play out in the news media, with various groups seeking to influence the discussion. This paper examines news coverage of recommendations to restrict alcohol advertising to see how supporters and opponents frame the debate, with a view to providing some suggestions for policy advocates to advance the discussion.

**Methods:**

We used content and framing analyses to examine 329 Australian newspaper items mentioning alcohol advertising restrictions over 24 months. All items were coded for mentions of specific types of advertising and types of advertising restrictions, the presence of news frames that opposed or endorsed advertising restrictions, statements made within each frame and the news-actors who appeared.

**Results:**

Restrictions were the main focus in only 36% of 329 items. Alcohol advertising was conceived of as television (47%) and sport-related (56%). Restrictions were mentioned in non-specific terms (45%), or specified as restrictions on timing and placement (49%), or content (22%). Public health professionals (47%) appeared more frequently than drinks industry representatives (18%). Five supportive news frames suggested the policy is *a sensible public health response*, *essential to protect children*, *needed to combat the drinks industry, required to stop pervasive branding*, or as *only an issue in sport*. Four unsupportive frames positioned restrictions as *unnecessary for a responsible industry, an attack on legitimate commercial activities, ineffective and ‘nannyist’,* or *inessential to government policy*. Support varied among news-actors, with public health professionals (94%) more supportive than the public (68%), community-based organisations (76%), the government (72%), and the sports (16%), drinks (3%), or advertising (4%) industries.

**Conclusion:**

Restrictions on alcohol advertising currently have low newsworthiness as a standalone issue. Future advocacy might better define the exact nature of required restrictions, anticipate vocal opposition and address forms of advertising beyond televised sport if exposure to advertising, especially among children, is to be reduced.

## Background

A significant proportion of Australians consume alcohol at levels risky to personal and public health and safety [1,2]. Accordingly, addressing problematic consumption of alcohol has been given high priority for action, with best practice recommending universal interventions that target the whole population, rather than intervening with just high-risk drinkers [3]. Such interventions aim to reduce net alcohol consumption, producing attendant reductions in alcohol-related harm, and are evaluated as cost-effective [3-5].

Thus, attention has focused on the need for policy reform of alcohol advertising and promotional activities [6-8], with emphasis on young people [9] and sport-related sponsorships and branding [10,11]. While there is considerable research estimating children’s and adolescents’ exposure to alcohol advertising in movies [12], television programming [13,14], magazines [15] and even student publications [16], there is still debate over the evidence regarding alcohol advertising’s relationship with consumption [17,18]. Research conducted in the 90s suggested no strong link between advertising expenditure and consumption patterns at a general population level [19,20]. However, among young people, increased exposure to alcohol advertising is positively associated with later consumption patterns [21-24], which can vary according to regional differences in advertising budgets [25]. A 2009 review of longitudinal studies found “consistent evidence to link alcohol advertising with the uptake of drinking among non-drinking young people, and increased consumption among their drinking peers” (p 242; [26]. Young people like alcohol advertisements, with likeability positively related to their intentions to purchase the advertised product [27]. While watching alcohol advertisements, young Australians report perceived messages “that alcohol consumption leads to social and other success, increases confidence and attractiveness…” (pg 350 [28] and alcohol advertising promotes sexual stereotypes [29].

Within this context, sustained calls to restrict alcohol advertising and regulate promotion using legislation have emerged in the public health community [5,30-34], who point to the success of advertising restrictions in tobacco control and the cost-effectiveness of partial bans or restrictions [5]. Unlike tobacco, not all use of alcohol is considered harmful, yet it has been argued that there are enough similarities that lessons from tobacco control could potentially be usefully adapted for alcohol [35].

Current restrictions on alcohol advertising in Australia are not legislated but are voluntary and self-regulated by the drinks industry via the Alcoholic Beverages Advertising Code (ABAC) [36] and the Australian Association of National Advertisers (AANA) Advertiser Code of Ethics [37]. These agreements state that alcohol advertisements must not, for example, encourage binge-drinking, or appeal to children, or link social and sexual success with alcohol consumption. For television broadcasts, advertising must also comply with the Commercial Television Industry Code of Practice (CTICP) [38], which prevents alcohol being directly advertised on television before 8.30 pm, yet a major exemption exists for live sports broadcasts. Complaints about alcohol advertisements can be made to the Advertising Standards Board (ASB). Advocates for greater regulation point out that internal documents from alcohol companies show advertising strategy is aggressive and runs counter to the spirit of self-regulated codes [39]. Other criticisms include that voluntary codes fail to prevent underage exposure [13]; companies deliberately target youth with promotional activities anyway [14]; there are numerous examples of non-compliance with existing guidelines [40-42]; and that in Australia, boards who review complaints about advertisements, do not agree with members of the general public [43] or independent experts [44], that alcohol advertisements have breached the guidelines in the voluntary codes. In most of these regards, similarities have been noted between the alcohol industry and the tobacco industry [35].

The Alcohol Working Group of the Australian Government’s National Preventative Health Taskforce (NPHT) recommended regulating alcohol promotion to reduce consumption [3,31]. These recommendations were largely in concert with those of the World Health Organisation [32] and confined to television advertising and event sponsorship, as follows: 

Advertising during live sports broadcasts

Advertising during high adolescent/child viewing

Sponsorship of sport and cultural events

"“In a staged approach, phase out alcohol promotions from times and placements which have high exposure to young people aged up to 25 years, including:"

"Monitor and evaluate the effectiveness of the voluntary approach to alcohol promotions"

"Introduce independent regulation through legislation if the co-regulatory approaches are not effective in phasing out promotions from times and placements which have high exposure to young people up to 25 years.”"

As elsewhere, alcohol control policies in Australia have been highly contested, with policies targeting the whole population rather than problem drinkers being unpopular [45,46] or only supported by specific groups in the community such as police, while licensees oppose the policies [47].

Debates about alcohol control policies regularly play out in Australian news media, and the way the various arguments are framed by participants in these debates is critical to how different audiences understand and evaluate the issues involved and who is responsible for change. According to Entman [48] “to frame is to select some aspects of a perceived reality and make them more salient in a communicating text, in such a way as to promote a particular problem definition, causal interpretation, moral evaluation, and/or treatment recommendation for the item described.” Framing leaves some aspects of issues absent or in the background, highlighting particular “preferred” ways of seeing [49]. Interest groups interacting with news media seek to assert their framings of the meaning of issues and incidents and to re-frame dominant narratives that have become established [50,51] and which are not conducive to legislative or policy reform. Considerations of newsworthiness, journalists’ personal values and the accessibility of spokespeople all affect story selection and how it is approached [50-52] as well as the ways in which different audiences interpret the meaning of news [53,54]. Thus, news frames can directly affect public perception and awareness of issues and influence health-related behaviour [55].

In alcohol control, an array of interest groups comment on policy proposals and vie for their interpretations to be heard and to dominate the way these issues are characteristically treated by media and understood by audiences [56,57]. Given the unpopularity of some alcohol-control policies as well as the high proportion of the population that consumes alcohol, news reports of such policies are bound to be highly contested. For health advocates who participate in public conversations regarding alcohol, recognition of factors that ultimately influence audiences’ evaluations is crucial to more strategic framing of policies as legitimate concerns and targets for reform.

Alcohol receives prominent news coverage in Australia [57,58] and in this paper we review coverage of proposals to restrict alcohol advertising and promotions before and after the release of recommendations in the NPHT’s report [31]. We were interested in the extent to which the NPHT report may have stimulated news coverage, how advertising restrictions were framed by news actors appearing in the news, who supported and opposed such restrictions, and whether these news frames reflected patterns that have been observed in the field of tobacco control.

## Methods

Using the Factiva news database, we reviewed news coverage and commentary on alcohol advertising and promotion in 13 Australian national and capital city newspapers for two 12 month periods immediately before and after September 1 2009, when the National Preventative Health Strategy was launched. We also examined all television news and current affairs coverage of the same issue on five free-to-air Sydney television stations over the same period, using the digital database of the Australian Health News Research Collaboration [59]. For newspapers, we searched news, features, editorials and letters to the editor. Items were identified using the search keywords: alcohol, advertising, promotion and policy. All items located were then viewed for the inclusion of any mention of restrictions on advertising or proposals to regulate its promotion. Items that mentioned alcohol, but not advertising policy were excluded, as were those that mentioned other alcohol-control policies unrelated to alcohol advertising.

We analysed coverage at two levels. The first was a descriptive content analysis of the news items [60], summarising the alcohol advertising and restrictions mentioned. The second level of analysis was guided by framing theory [48,49,61] and identified particular news-frames used within each article to explain any proposal to restrict or regulate alcohol advertising. Details of each analysis are expanded below.

Primary coding was completed by author AF and inter-coding reliability was assessed by a second coder (SH) via two coding exercises. The first exercise examined inclusion or exclusion of 25 randomly generated items to test the reliability of decisions regarding excluded items. A second exercise assessed reliability of framing decisions on a randomly generated sample of 15% of all statements recorded.

### Content analysis

A content coding sheet was developed and trialled on 20 news items outside of the study period. We defined alcohol advertising as including traditional media (e.g. television advertisements), new media (e.g. viral marketing), and promotional activities (e.g. sports sponsorship) [4,31].

The following variables were coded for each item:

Main focus: whether advertising restrictions were the main, or only a secondary focus of the item

Forms of advertising: whether advertising was referred to in general terms or whether specific kinds of advertising was mentioned (e.g. television ads or sporting sponsorship)

Restrictions mentioned: whether restrictions were mentioned in general terms, or whether certain types of restriction were specified (e.g. restrictions on advertising content or restrictions on the frequency or placement of advertisements)

Advertising organisations: whether mention was made of advertising and/or marketing organisations or peak bodies administering advertising codes

ABAC scheme: whether any item mentioned the drinks industry’s voluntary advertising code

NPHT reports: whether the items mentioned the National Preventative Health Taskforce’s report [3] or its alcohol working group’s technical report [31]

News-actors present: Recorded the presence of the following news-actors: government representatives, public health professionals, drinks industry, advertising industry, law enforcement, non-government and community-based organisations, reporters, general public (*vox populi)*

### Framing

Following Terkildsen et al. [50], articles were coded for salient issues relevant to restrictions on alcohol advertising and whether the frame was supportive or unsupportive of the proposed restriction. News frames were defined by arguments made for or against introducing greater restrictions on alcohol advertising, with particular outcomes either stated or implied (e.g. lower consumption, no effect, etc.) by the coverage.

Within each frame, we recorded statements and coded which news-actors from particular interest groups made the statement. A statement was any direct quote (X said “Y”) or attribution (“X said that…”) by a news-actor or a direct argument made by a journalist. We assessed the distribution of identified frames across the coverage and support for alcohol advertising restrictions within interest groups.

## Results

Our broad search strategy returned 1,101 newspaper items. Of those, 70.1% (n = 772/1,101) were excluded, as described above. We also found only eight television news reports mentioning alcohol advertising restrictions. Given the very low volume of relevant television reports, we focussed only on newspaper coverage for our detailed results. 329 newspaper articles were therefore used as the basis for this study. Of those 329 articles, 186 occurred in the year before (hereafter ‘pre’) the release of the National Preventative Health Strategy and 143 in the year after (hereafter ‘post’) the release.

Assessment of inter-coder reliability using Cohen’s Kappa [62] produced scores of 0.92 for inclusion criteria and 0.74 for coding of statements indicating excellent and good agreement respectively [60,62,63].

Note: during the analysis we discovered that in the year preceding the release of the National Preventative Health Strategy, 27.4% (n = 51/186) of newspaper articles mentioned alcohol advertising restrictions only in passing in relation to a particular policy debate about. At the time, the ‘alcopops tax’ [57], which aimed to increase the tax rate on pre-mixed alcoholic drinks, was being debated in parliament and one independent Senator initially made his support for the tax conditional upon the government introducing legislation to restrict television advertising of alcohol during live sports broadcasts. Mention of advertising restrictions in this context consisted of only one or two sentences, while the majority of each article focused on the tax. Due to the often-reported Senator’s concern for breaking links between alcohol advertising and sport, we have included these articles in the analyses as an example of how policies are discussed publicly. However, we contend that without the pre-existing media interest in the contested alcopops tax, alcohol advertising restrictions might never have received this level of coverage. Accordingly, in results tables, we report data for the “pre” period in two ways: (i) all newspaper articles included and (ii) all newspaper articles, excluding those articles related to the alcopops tax.

We report data in text for the full 24 months of coverage. Full pre and post figures are found in tables.

### Main focus of news items

Advertising restrictions were the main focus in 35.9% (118/329) of articles on alcohol. Where they were not the main focus, restrictions were situated within broader contexts such as items on alcohol and associated harms in general (18.5%; 61/329), or prevention of diseases related to tobacco, obesity and alcohol (11.6%; 38/329). (Table 1).

**Table 1:** Summary - mentions of specific advertising channels, types of advertising restrictions and the news-actors present in each story before and after the Preventative Health Taskforce Report

		**PRE (n=186)**	**POST (n=143)**	**PRE EXCL. ALCOPOPS (n=135)**
		**%**	**%**	**%**
**Type of advertising discussed**				
	Sport – general	64.0	56.6	46.2
	Television advertising	53.8	49.3	37.8
	Point-of-sale promotions (e.g. happy hour, discounted alcohol)	12.4	16.9	20.3
	Sport – specific teams (e.g. cricket, AFL)	16.7	21.3	16.8
	Festivals/cultural events (e.g. ‘schoolies’ week, Big Day Out)	2.7	3.7	12.6
	Internet, social network websites and online viral marketing	13.5	18.4	10.5
	Public space advertising (e.g. billboards, public transport)	12.4	16.9	9.8
	Cinema advertising and product placements	7.0	9.6	4.9
	Radio advertisements	8.6	11.0	4.2
	OTHER (e.g. video games, music videos, information booklets)	5.4	6.6	4.9
**Types of advertising restrictions or regulations**				
	Unspecified, general	41.9	46.3	49.0
	Reducing frequency of and exposure to advertising	55.4	48.5	40.1
	Total ban on all forms of advertising	19.9	26.5	14.0
	Restrictions on content	18.3	22.1	14.0
	OTHER	2.7	3.0	18.9
**News-actors present**				
	Public health professional	44.1	55.1	51.7
	Government representative	52.7	39.0	37.8
	Liquor industry	16.7	15.4	20.3
	*Vox populi*	16.7	20.6	18.2
	Journalist	19.9	22.1	16.8
	Advertising and/or marketing	6.9	9.5	11.2
	Non-government or community-based organisation	11.3	13.2	6.3
	Law and order	0.5	0.7	6.3
	Other	10.2	14.0	6.3

Note: categories are not mutually exclusive.

### Types of advertising mentioned

The most commonly mentioned form of advertising was advertising or promotion related to sport (56.2%; 252/329). This included concerns like sponsorship of teams or events, advertising during live broadcasts and field banner advertising. After sport, the focus was most commonly on television advertising (46.8%; 154/329) and point-of-sale promotions (15.8%; 52/329). Additionally, though mentions of promotion at festivals and cultural events was small overall, there was a significant increase in mentions of such advertising between the first and second year of coverage (pre 2.7% versus post 12.6% p < .001;) (Table 1).

### Type of restrictions proposed

Just under half of all items (45%; 148/329) mentioned ‘advertising restrictions’ without stating what kind of restrictions they meant. A higher proportion (48.9%; 161/329) of items mentioned specific restrictions on timing and placement of alcohol advertisements (note, these are not mutually exclusive categories)(Table 1). While mentions of each kind of restriction were similar between the two time periods, the proportion of articles referring to restrictions on timing and placement significantly decreased in the second year of coverage (p < .001).

### News actors

Public health professionals were the most frequent news actors (47.4%; 156/329), followed by government representatives (46.2%; 152/329), the drinks industry (18.2%; 60/329) and the general public (17.3%; 57/329). Less common were representatives of the advertising industry, non-government and community-based organisations, and police, lawyers or judges. (Table 1).

A small proportion of articles (8.8%; 29/329) mentioned the Alcohol Beverages Advertising Code and only 11.2% (37/329) referred to advertising organisations and peak bodies such as the Advertising Standard Bureau, the Australian Association of National Advertisers, or the Australian Communication and Media Authority. During the second year of coverage, one third of articles (33.6%; 48/143) mentioned the NPHT’s reports.

### News-framing of alcohol advertising restrictions by interest groups

From 329 news articles, 1,322 statements (pre n = 814; post n = 508) were identified which were relevant to alcohol advertising restrictions and formed the basis of our framing analyses. We identified ten prominent news frames that accounted for the majority of coverage. Five frames were supportive of advertising restrictions, four unsupportive and one neutral, or voicing new ideas about the current policy proposal (Table 2). Table 3 shows the proportion of each news actor group that was supportive or unsupportive of advertising restrictions.

**Table 2:** Distribution of different frames across the coverage (N = 1,322 statements)

	**PRE**		**POST**		**PRE EXCL. ALCOPOPS**	
	**n**	**%**	**n**	**%**	**n**	**%**
**SUPPORTIVE FRAMES: Advertising restrictions as….**						
· The sensible public health response e.g. “*“First, the Government needs to ban alcohol advertising, especially on television, as was done for tobacco”*	139	17.1	103	20.3	119	16.9
· Necessitated by the disingenuous drinks industry e.g. “*…industries will never agree to effective controls on their irresponsible promotions”*	78	9.6	77	15.2	76	10.8
· Indispensable counter to pervasive advertising culture e.g. *“Now I have seen it all, an Australian Digger and Victoria Cross winner used to market beer”*	63	7.7	45	8.9	61	8.7
· Crucial in sport e.g. *“four out of five people wanted to see an end to alcohol sponsorship in all local sports clubs, provided there were funds to replace the lost revenue.”*	178	21.9	38	7.5	132	18.8
· Necessary protection for children e.g. “*Should alcohol advertising be banned? No, certain types should be, such as those that particularly target young people. I'm a wine drinker so I like to learn about different varieties from their ads.”*	62	7.6	34	6.7	59	8.4
· **Total supportive**	**520**	**63.9**	**297**	**58.5**	**447**	**63.5**
· **UNSUPPORTIVE FRAMES: Advertising restrictions as…**						
· Overkill and unwarranted for a responsible drinks industry that contributes to the community e.g. *“In terms of responsibility, we are absolutely like any other promoter out there in ensuring that we're doing everything we can…”*	180	22.1	92	18.1	163	23.2
· Pointless, ineffective, politically unfeasible and nannyist e.g. “*it's our right to rejoice in the pleasures of Aussie family life and mateship over a drink or two, and we should resent having that right trampled by do-gooder politicians and nanny-state troopers”*	49	6.0	37	7.3	47	6.7
· An attack on commercial freedom, creativity and jobs e.g. “*This is not the sort of policy a government would want to impose on struggling media companies in the middle of a major global economic recession”*	28	3.4	32	6.3	26	3.7
· Non-urgent and not government’s preferred policy e.g. *“While the government is supportive of limiting the exposure of children to advertising that may unduly influence them, the government will not consider regulatory action at this time”*	29	3.6	10	2.0	18	236
· **Total unsupportive**	**294**	**36.1**	**171**	**33.7**	**254**	**36.1**
· **NEUTRAL FRAMES**						
· New ideas e.g. *“Junk food and alcohol advertisements should be hit with a levy to force companies to market less harmful products”*	-	-	27	5.3	-	-
· Neutral	7		13	2.5	3	0.4
**TOTAL**	**814**	**100.0**	**508**	**100.0**	**704**	**100.0**

**Table 3:** News-actor support for advertising restrictions – statements N = 1,322

	**Public health**		**Government**		***Vox populi***		**Drinks industry**		**Advertising**		**Reporter**		**NGO or CBO**		**Sport**	
	**N**	**%**	**n**	**%**	**N**	**%**	**N**	**%**	**n**	**%**	**N**	**%**	**n**	**%**	**n**	**%**
**Supportive news frames**																
A sensible public health response	113	29.5	56	25	30	18.1	-	-	3	3.1	30	12.9	9	23.7	1	1.2
Essential to protect the young	52	13.6	5	2.2	16	9.6	1	1.0	-	-	19	8.2	2	5.3	1	1.2
Combat disingenuous industries	79	20.6	20	8.9	19	11.4	1	1.0	1	1.0	27	11.6	6	15.8	2	2.4
Essential control on pervasive branding	46	12.0	9	4.0	24	14.5	1	1.0	-	-	20	8.6	7	18.4	1	1.2
Especially necessary in sport	70	18.3	71	31.7	24	14.5	-	-	-	-	38	16.4	5	13.2	8	9.6
**Unsupportive news frames**																
Unnecessary for a responsible industry	1	0.3	5	2.2	8	4.8	92	93.9	38	38.8	56	24.1	8	21.1	64	77.1
An attack on legitimate commercial activity	-	-	-	-	-	-	2	2.0	50	51.0	6	2.6	-	-	2	2.4
Ineffective and ‘nannyist’	2	0.5	7	3.1	42	25.3	-	-	4	4.1	26	11.2	1	2.6	4	4.8
Seen as unnecessary by the government	1	0.3	36	16.1	1	0.6	-	-	-	-	1	0.4	-	-	-	-
**NEUTRAL FRAMING**	19	5.0	15	6.7	2	1.2	1	1.0	2	2.0	9	3.9	-	-	-	-
**TOTAL**	**383**	**100**	**224**	**100**	**166**	**100**	**98**	**100**	**98**	**100**	**232**	**100**	**38**	**100**	**83**	**100**

### Supportive news frames

#### Advertising restrictions as a sensible public health response

This frame depicted advertising restrictions as a sensible societal response to problematic alcohol consumption, emphasising the policy as part of a comprehensive package of policies such as increased taxation, shorter trading hours and stronger policing of alcohol related violence. Restrictions were positioned as effective tools in a larger suite of preventive policies and practices. Such framing echoed a larger preventive health context, often discussing alcohol as part of a ‘big three’ set of problems: alcohol, tobacco and obesity. Advertising restrictions were promoted as effective and feasible, given the success of similar policies in tobacco control, where it was implied that lessons could be adapted for alcohol. Sometimes such framing would refer to the case for advertising restrictions as self-evident and obvious, without further justification.

#### *Advertising restrictions as essential to protect the young-"think of the children"*

Advertising restrictions were often framed as concern for youth: as something from which more vulnerable members of society should be protected. It was often illustrated by indignant claims about targeting children such as placing billboard advertisements near schools or sponsoring music festivals. Such framing accused the drinks industry of deliberately using characters and promotional material that would appeal to children and young people, for example the anthropomorphic “Bundy Bear” rum advertising or decorated hipflasks sold in a store frequented by girls. These items often included demands that greater regulation of advertising to young people be implemented immediately.

#### Advertising restrictions necessary to combat disingenuous industries

This frame saw restrictions on alcohol advertising as essential to counteract the behaviour of the drinks and advertising industries, which were positioned as variously insincere, duplicitous and dishonest. Such framing was largely cynical about the value of industry self-regulation, emphasised the size of advertising budgets and the conflict of interest between reductions in advertising and commercial imperatives to maximise profit. The notion of corporate responsibility for harms associated with their products was often expressed.

#### Advertising restrictions as essential to controlling pervasive branding and promotion

Here, alcohol advertising was characterised as pervasive across cultural events, relentless in its frequency and messaging, constantly ‘pushing the limit’ by promoting socially acceptable stereotypes of drinking as normative. For example, the pairing of a “raise a glass” campaign with the Returned Serviceman’s League on ANZAC day was seen as alcohol advertising infesting yet another iconic cultural space. Branding and promotional activities were framed as wielding too great an influence in public spaces and as inescapable in a 24/7 culture. Advertising restrictions were thus positioned as a vital and necessary counter to the power of advertising and the emphasis here was not so much on achieving health goals, as on reducing the scope, frequency and ubiquity of advertising.

#### Advertising restrictions necessary in sport

This news frame positioned alcohol advertising as problematic mostly in relation to sport, whether professional sporting codes (e.g. cricket, rugby) or local community-based groups with historical reliance on funding from the drinks industry. Support for restrictions in this frame was articulated as the need to end messages linking sporting success with alcohol. This frame emphasised that television alcohol advertising curfews were ineffective when exemptions were made for live sports broadcasts and that something should be done about this. This framing focused on changing attitudes towards sporting success and acceptance of alcohol’s place within the sporting arena.

### Unsupportive news-frames identified

#### Advertising restrictions: unnecessary for a responsible industry

In this news frame, advertising restrictions were positioned as unwarranted by a responsible drinks industry that was said to be already actively managing alcohol risk. Such framing emphasised existing guidelines as more than adequate, raised examples of the industry reacting swiftly to complaints and policing its own promotional material, denied that the industry caused harm directly or targeted children and stressed their importance to community as funders of events. This angle sought to re-frame the public health position on advertising restrictions as unnecessary punishment of moderate drinkers for the behaviour of a few people and cheap political point-scoring at the industry’s expense.

#### Advertising restrictions as an attack on legitimate commercial activity

This frame suggested that introducing greater regulation of alcohol advertising would be an attack on the advertising industry. Negative consequences such as job losses, erosion of commercial freedom, the stifling of creativity and negative impact on the economy were highlighted. Such framing included calls to lobby the government directly to oppose the policy. No mention was made of alcohol-associated harms and supporters of restrictions were derided as seeking a “quick fix”.

#### *Restrictions as ineffective and "nannyist"*

Here, advertising restrictions were deemed ill-conceived and ineffective. This was often taken to be self-evident, with no argument advanced. Where explanation was offered, the policy was dismissed as poorly-targeted and statements asserted that alcohol advertising does not affect consumption and that consumption was more proximally influenced by other factors, like price. Such framing predicted that that the policy would be automatically rejected by the public as an example of the “nanny state” needlessly interfering with people’s choices. In keeping with this assertion, members of the public often stated that the government was too bound by vested interests or political donations to even consider it, regardless of whether they personally supported or opposed the policy.

#### Government re-framing restrictions as unnecessary

This frame occurred in a relatively small proportion of coverage, as it was dependent on two particular incidents. During the first year of coverage, an independent Senator advocated strongly for the legislation of advertising restrictions in return for supporting the government’s alcopops tax. This was rejected by the government. Similarly, during the second year of coverage, the government responded to the NPHT suggestions regarding alcohol advertising and again rejected legislating changes, electing instead to pursue a “voluntary and collaborative” approach with the drinks industry [64]. Thus, for a short period in each year, the government positioned advertising restrictions as non-urgent.

### Neutral framing and new ideas

A small proportion of statements on alcohol advertising did not either support or oppose advertising restriction (for example, a suggestion about creating a levy on the advertising budgets of the drinks industry to be used variously to fund treatment services, counter-advertising educating people about the harms of alcohol).

### Interest groups and news actor support for advertising restrictions

Table 3 shows the number of statements made by each news actor group within news frames that supported or opposed advertising restrictions. A majority of statements made by public health news-actors (93.7%); the public (68.1%), members of non-government or community based-organisations (76.4%) and government representatives (71.9%), were supportive of restrictions on alcohol advertising while nearly all statements made by representatives of the drink industry (95.9%), the advertising industry (93.9%) or sporting organisations (84.3%) were unsupportive.

Public health actors were most likely to frame arguments supporting advertising restrictions as sensible public health (29.5%) or a necessary response to the disingenuous drinks and advertising industries (20.5%), while government representatives were more likely to frame their support around concern about alcohol advertising in sport (31.7%). Although members of the public supported restrictions on the whole, the most frequently deployed news-frame was an argument that policies to restrict advertising are ‘nannyist’ (25.3%). News-actors in the drinks industry emphasised their already responsible industry (93.9%), as did sporting organisations (77.1%), while representatives of the advertising industry most frequently emphasised their commercial activities (51.0%) as legitimate enterprises.

## Discussion

In contrast to Australia’s comprehensive ban on tobacco advertising that has been incrementally implemented since September 1976, in 2012 there are effectively no legislative controls on alcohol advertising beyond industry voluntary agreements. Unlike several of the Taskforce’s key recommendations on tobacco control which the government implemented [65], those on restricting alcohol promotions with legislation have been rejected in favour of continued monitoring, with annual reporting back to the Minister [64]. The task for alcohol control advocates therefore remains imposing if the goal is legislating necessary restrictions on alcohol advertising.

Media advocacy for legislation on advertising restrictions will be essential in building public and political support for legislative change and will inevitably meet with protracted opposition [66]. Our study provides the first analysis of reportage of such advocacy and reaction to it, in a context where some of the clearest and strongest recommendations have been publicly stated regarding alcohol advertising. Our primary finding is that this issue currently has low newsworthiness as a standalone issue. We found minimal coverage of proposals to restrict alcohol advertising on five Sydney free-to-air television channels over 24 months, and only 329 newspaper mentions in 13 leading newspapers. Of these, about two thirds situated advertising restrictions within a broad context, where restrictions alone were not the sole focus of reportage or the top priority where many alcohol control policies were discussed. Thus, while it is a major policy concern for public health professionals in the alcohol sector, it cannot yet be said to be a major news focus in Australia.

We had hypothesised that examining coverage before and after the release of the NPHT’s policy recommendations for alcohol would demonstrate greater debate concerning restrictions on alcohol advertising. Yet in the 12 months after the report, we did not see any increased reporting about alcohol advertising after the release of the policy and thus, unlikely to impact on the public’s awareness of the issue. In the absence of news-production studies, we cannot be certain why it did not attract greater news coverage, but suspect that a focus on other recommendations from the report, in combination with the government’s failure to support change may have contributed. This may have seen policy reform advocates loathe to be publicly critical of a government they hoped may act later, judging that little would be gained by such criticism. Perhaps reflecting this, we found very few news reports where alcohol control advocates were openly critical of government, a sentiment that was expressed more often by members of the public.

Our second main finding is that there is a general lack of specificity in the coverage about what alcohol advertising encompasses and exactly what policy reform advocates would like to see changed in the future. While alcohol experts and advocates may be clear on these distinctions and priorities, the detailed nuances are yet to be reflected clearly in newspaper coverage that discusses the issue. Advertising was mostly referred to in general terms, as if it were a single entity. Few distinctions were made evident between the large range of traditional advertising media and the more non-traditional promotional activities engaged in by the alcohol industry (e.g. social media pages, festival sponsorships, merchandise giveaways etc.). The few times it was specified, alcohol advertising was largely characterised as ‘on television’ and ‘of concern for children’, which fails to consider the wider opportunities for exposure to alcohol advertising. For example, there was little acknowledgment of the internet with its social networks. Current research shows that alcohol branding activity on major social networks includes interactive games and suggestions to drink [67], while some young users of social networks present alcohol as a major component of their identity, which correlates to problematic consumption [68]. Neither the NPHT recommendations, nor the newspaper coverage reported here showed any great focus on the issue, an important omission when online marketing opportunities and viral marketing are likely prove significant barriers to reducing people’s exposure to advertising [67]. Future advocacy might expand the discussion of the different avenues used for alcohol promotion, usefully highlighting the limitations of television curfews when underage audience members are likely to be exposed elsewhere, regardless.

While the present low level of news coverage mostly features voices supportive of advertising restrictions, the lack of specificity in these reports suggests that advocacy experts have not always expressed the same vision, or been reported by journalists as in agreement. We found no consistent articulation of precisely what changes advocates sought: some prioritised government legislation concerning content of advertisements, while others prioritised timing and placements of advertisements and so on. There was some agreement that children and young people were a high priority, yet news reports were not clear that the sector was in agreement over where to start.

Indeed, though the NPHT report makes clear recommendations about focusing initial reform on underage audiences and advertising in sport, together these news frames only accounted for only 32% of all statements made by public health representatives. While universal agreement among health experts is not a pre-requisite, we suggest this is a clear opportunity for future advocacy regarding agreed policy recommendations.

As with previous studies [57,69], the majority of news reports featured commentary from public health experts. Coalitions of these voices that provide comment on policy reform [33,70], have focused on alcohol marketing. While the volume of news coverage on alcohol promotion restrictions has been modest, advocates might take some encouragement from the majority of statements being supportive of restrictions. The dominant way of reporting is to frame it supportively, with recognition of the underlying health imperatives clear in the coverage. Should the issue gain greater levels of political traction though, those opposing restrictions could be expected to increase their profile, and to focus on specific proposals as well as locating their critiques within general negative framings about the economy and the “nanny state” as was the case with tobacco control and the ‘alcopops’ tax [57], and demonstrated in this paper. In the second year of coverage in our data, we noted a greater proportion of negative statements made by those in the advertising industry, as well drinks industry representatives continually emphasising they were already responsible. Future policy advocacy should thus anticipate further vocal, public opposition to the policy from this sector and consider how they would respond to the arguments documented here that job security and commercial freedoms are threatened by such restrictions. Monitoring, critically evaluating and planning strategic responses to such opposition will be of critical importance if any advances are to be made; this occurred with advocacy for tobacco advertising controls and was crucial to ensuring their successful introduction over time. We acknowledge that tobacco control sought to ban all tobacco advertising, which is not recommended for alcohol and our results likely reflect the complexity in responding to alcohol advertising that covers a broad range of mediums and different audiences.

We suggest that given this complexity, and the present lack of governmental support for legislative changes, future advocacy could further emphasise the failure of existing structures to regulate alcohol advertisements. The systems of alcohol advertising self-regulation through voluntary codes of ethics that today substitute for legislative controls received barely any news coverage. While public health professionals are aware of the Alcoholic Beverage Advertising Codes and their inadequacies [41,44], the issue has not yet become newsworthy, even with strong recommendations in the NPHT report. Currently, newspaper readers are likely to be unaware a self-regulatory code even exists, or that complaints about alcohol advertising can be made on this basis. Perhaps recognising this, some public health agencies have recently formed the Alcohol Advertising Review Board (AARB) [71], an alternative mechanism to consider and publicise complaints about alcohol advertising. This may prove to be a vehicle that will enable the shortcomings of self-regulation to receive publicity and lead to the inevitable “what needs to be done?” news narrative that forces consideration of change. Our suggestion for future advocacy is ongoing promotion of the awareness of the codes, how to make complaints, the need to make complaints and the two boards (ASB and AARB) that provide avenues of complaint. While we acknowledge this process functions in a space where exposure to alcohol advertising has already occurred, it nevertheless represents opportunity for advocacy in the absence of legislated changes to self-regulation.

Lastly, we found that where members of the public were reported in the coverage, they were largely supportive of alcohol advertising restrictions. This was especially true with regard to sport-related promotions, echoed by other research [72]. Future advocacy efforts might benefit from careful consideration of how to capitalise on existing sentiment, should alcohol advertising restrictions gain greater traction both politically and publicly in news reporting.

## Conclusion

In conclusion, we see that while current news coverage of alcohol advertising restrictions is largely positive and recognises health benefits behind such a policy, there is still a need to further promote the policy to increase its newsworthiness and interest to both the public and policy makers. Advocates might consider defining more precisely the kinds of restrictions they would like to see prioritised, speaking mostly about those priorities in the news and address other forms of advertising beyond televised sport that still have the potential to be seen by underage children. Given existing opportunities for public health professionals to comment in the news, advocates should anticipate further vocal opposition from interest groups and prepare their response accordingly. Future research should focus on how news media messages about alcohol policies are received by audience members.

## Competing interests

The authors have no competing interests to declare.

## Authors' contributions

ASF conceived of the study, participated in its design, coded all data, performed statistical analysis and drafted the manuscript. SC participated in the design of the study, oversaw data coding and analysis and helped draft the manuscript. Both authors read and approved the final manuscript.

## Authors' information

All authors are members of the Australian Health News Research Collaboration and invite collaboration with any health researchers interested in news media reporting of health problems. For details see here: http://sydney.edu.au/medicine/public-health/AHNRC/.

## Pre-publication history

The pre-publication history for this paper can be accessed here:

http://www.biomedcentral.com/1471-2458/12/727/prepub

## References

[B1] AIHW 2010 National Drug Strategy Household Survey report. Drug statistics series no. 25. Cat. no. PHE 145 2011 AIHW, Canberra [accessed on 11th November 2011]. Available from: http://www.aihw.gov.au/publication-detail/?id=32212254712

[B2] Laslett A-M Room R Ferris J Wilkinson C Livingston M Mugavin J Surveying the range and magnitude of alcohol's harm to others in Australia Addiction 2011 106 9 1603 1611 10.1111/j.1360-0443.2011.03445.x 21438943

[B3] National Preventative Health Taskforce Australia: the healthiest country by 2020 2009 National Preventative Health Strategy - the roadmap for action. Australian Government Department of Health and Ageing, Canberra, ACT

[B4] Babor T Caetano R Casswell S Edwards G Giesbrecht N Graham K Alcohol: no ordinary commodity research and public policy 2010 2 Oxford University Press, Oxford

[B5] Collins DJ Lapsley HM The avoidable costs of alcohol abuse in Australia and the potential benefits of effective policies to reduce the social costs of alcohol 2008 Canberra, Australia

[B6] Jernigan DH Framing a public health debate over alcohol advertising: The Center on Alcohol Marketing and Youth 2002–2008 J Public Health Policy 2011 32 2 165 179 10.1057/jphp.2011.5 21346788

[B7] Meier PS Alcohol marketing research: the need for a new agenda Addiction 2011 106 3 466 471 10.1111/j.1360-0443.2010.03160.x 21299664

[B8] Casswell S Current status of alcohol marketing policy—an urgent challenge for global governance Addiction 2012 107 3 478 485 10.1111/j.1360-0443.2011.03701.x 22313102

[B9] Jernigan DH Jernigan DH Importance of reducing youth exposure to alcohol advertising Arch Pediatr Adolesc Med 2006 160 1 100 102 10.1001/archpedi.160.1.100 16389218

[B10] Kelly B Baur LA Bauman AE Smith BJ Saleh S King LA Role modelling unhealthy behaviours: food and drink sponsorship of peak sporting organisations Health Promot J Austr 2011 22 1 72 75 21717842 10.1071/he11072

[B11] Jones S When does alcohol sponsorship of sport become sports sponsorship of alcohol? A case study of developments in sport in Australia Int J Sports Mark Sponsorship 2010 11 3 250 261

[B12] Dal Cin S Worth KA Dalton MA Sargent JD Youth exposure to alcohol use and brand appearances in popular contemporary movies Addiction 2008 103 12 1925 1932 10.1111/j.1360-0443.2008.02304.x 18705684 PMC3541777

[B13] Fielder L Donovan RJ Ouschan R Exposure of children and adolescents to alcohol advertising on Australian metropolitan free-to-air television Addiction 2009 104 7 1157 1165 10.1111/j.1360-0443.2009.02592.x 19438841

[B14] Winter MV Donovan RJ Fielder LJ Winter MV Donovan RJ Fielder LJ Exposure of children and adolescents to alcohol advertising on television in Australia J Stud Alcohol 2008 69 5 676 683 10.15288/jsad.2008.69.676 18781242

[B15] King C Siegel M Jernigan DH Wulach L Ross C Dixon K Adolescent exposure to alcohol advertising in magazines: an evaluation of advertising placement in relation to underage youth readership J Adolesc Health 2009 45 6 626 633 10.1016/j.jadohealth.2009.03.012 19931836

[B16] Cousins K Kypri K Alcohol advertising in the New Zealand university student press Drug Alcohol Rev 2008 27 5 566 569 10.1080/09595230802245246 18696302

[B17] Nelson JP Alcohol marketing policy: the missing evidence. Addiction 2012 107 9 1708 1709 10.1111/j.1360-0443.2012.03947.x 22861680

[B18] Casswell S Alcohol marketing policy: the missing evidence – a response to Nelson Addiction 2012 107 9 1709 1710 10.1111/j.1360-0443.2012.03965.x 22861680

[B19] Duffy M Advertising and the consumption of tobacco and alcoholic drink: a system-wide analysis Scot J Polit Econ 1991 38 4 369 385 10.1111/j.1467-9485.1991.tb00325.x

[B20] Nelson JP Broadcast advertising and US demand for alcoholic beverages Southern Econ J 1999 65 4 774 790 10.2307/1061275

[B21] Collins RL Ellickson PL McCaffrey D Hambarsoomians K Early adolescent exposure to alcohol advertising and its relationship to underage drinking J Adolesc Health 2007 40 6 527 534 10.1016/j.jadohealth.2007.01.002 17531759 PMC2845532

[B22] Stacy AW Zogg JB Unger JB Dent CW Exposure to televised alcohol ads and subsequent adolescent alcohol use Am J Health Behav 2004 28 6 498 509 15569584 10.5993/ajhb.28.6.3

[B23] Jones S Magee C Exposure to alcohol advertising and alcohol consumption among Australian adolescents Alcohol Alcohol 2011 46 5 630 637 21733835 10.1093/alcalc/agr080

[B24] Hastings G Anderson S Cooke E Gordon R Alcohol marketing and young peoples drinking: a review of the research J Public Health Policy 2005 26 3 296 311 10.1057/palgrave.jphp.3200039 16167558

[B25] Snyder LB Milici FF Slater M Sun H Strizhakova Y Effects of alcohol advertising exposure on drinking among youth Arch Pediatr Adolesc Med 2006 160 1 18 24 10.1001/archpedi.160.1.18 16389206

[B26] Anderson P de Bruijn A Angus K Gordon R Hastings G Impact of alcohol advertising and media exposure on adolescent alcohol use: a systematic review of longitudinal studies Alcohol Alcohol 2009 44 3 229 243 19144976 10.1093/alcalc/agn115

[B27] Chen MJ Grube JW Bersamin M Waiters E Keefe DB Alcohol advertising: what makes it attractive to youth? J Health Commun 2005 10 6 553 565 10.1080/10810730500228904 16203633

[B28] Jones SC Gregory P Munro G Adolescent and young adult perceptions of Australian alcohol advertisements J Subst Use 2009 14 6 335 352 10.3109/14659890802654524

[B29] Austin EW Hust SJ Targeting adolescents? The content and frequency of alcoholic and nonalcoholic beverage ads in magazine and video formats November 1999-April 2000 J Health Commun 2005 10 8 769 785 10.1080/10810730500326757 16316938

[B30] Doran CM Hall WD Shakeshaft AP Vos T Cobiac LJ Alcohol policy reform in Australia: what can we learn from the evidence? Med J Aust 2010 192 8 468 470 20402613 10.5694/j.1326-5377.2010.tb03589.x

[B31] National Preventative Health Taskforce Preventing alcohol-related harm in Australia: a window of opportunity. Australia: the healthiest country by 2020 2009 Alcohol Working Group, Australian Government, Canberra

[B32] World Health Organisation Global Strategy to Reduce the Harmful Use of Alcohol 2010 [accessed on 15/11/2011]. Available from: http://www.who.int/substance_abuse/alcstratenglishfinal.pdf

[B33] Alcohol Policy Coalition A nation drowning: the need for national alcohol reform 2010 [accessed on 11/11/2011]. Available from: http://117.55.235.172/attachments/123_ADF_Flyer_Fed_Individual_.pdf

[B34] Public Health Association of Australia Alcohol Policy 2008 [accessed on 11/11/2011]. Available from: http://www.phaa.net.au/documents/policy/20081006revisedAlcohol.pdf

[B35] Daube M Alcohol and tobacco Aust NZ J Publ Heal 2012 36 2 108 110 10.1111/j.1753-6405.2012.00855.x 22487342

[B36] The ABAC Scheme Alcohol beverages advertising (and packaging) code 2010 Avail:http://www.abac.org.au/uploads/File/ABAC%20Code%20at%206%20July%202010.pdf [accessed: 16/02/2010]

[B37] Australian Association of National Advertisers AANA Code of Ethics 2009 http://www.aana.com.au/documents/AANACodeofEthicsAugust2009.pdf accessed: 16/02/2012

[B38] FreeTV Australia The Commercial Television Industry Code of Practice 2010 [accessed on 29/05/2012]. Available from: http://www.freetv.com.au/media/Code_of_Practice/2010_Commercial_Television_Industry_Code_of_Practice.pdf

[B39] Hastings G They'll drink bucket loads of the stuff": An analysis of internal alcohol industry advertising documents. The Alcohol Education and Research Council 2009 Available:http://alcoholresearchuk.org/downloads/finalReports/AERC_FinalReport_0071.pdf [accessed: 16/02/2012]

[B40] Scott MM Cohen DA Schonlau M Farley TA Bluthenthal RN Alcohol and tobacco marketing - Evaluating compliance with outdoor advertising guidelines Am J Prev Med 2008 35 3 203 209 10.1016/j.amepre.2008.05.026 18692735 PMC2920147

[B41] Donovan K Donovan R Howat P Weller N Magazine alcohol advertising compliance with the Australian Alcoholic Beverages Advertising Code Drug Alcohol Rev 2007 26 1 73 81 10.1080/09595230601037026 17364839

[B42] Jones SC Lynch M Non-advertising alcohol promotions in licensed premises: does the Code of Practice ensure responsible promotion of alcohol? Drug Alcohol Rev 2007 26 5 477 485 10.1080/09595230701494390 17701510

[B43] Jones SC Donovan RJ Self-regulation of alcohol advertising: is it working for Australia? J Public Aff 2002 2 3 153 165 10.1002/pa.105

[B44] Jones SC Hall D Munro G Jones SC Hall D Munro G How effective is the revised regulatory code for alcohol advertising in Australia? Drug Alcohol Rev 2008 27 1 29 38 10.1080/09595230701499175 18034379

[B45] Tobin C Moodie AR Livingstone C A review of public opinion towards alcohol controls in Australia BMC Publ Health 2011 11 1 58 10.1186/1471-2458-11-58 PMC3048532 21272368

[B46] Wilkinson C Room R Livingston M Mapping Australian public opinion on alcohol policies in the new millennium Drug Alcohol Rev 2009 28 3 263 274 10.1111/j.1465-3362.2009.00027.x 21462409

[B47] Hawkins N Sanson-Fisher R Shakeshaft A Webb G Differences in licensee, police and public opinions regarding interventions to reduce alcohol-related harm associated with licensed premises Aust NZ J Publ Heal 2009 33 2 160 166 10.1111/j.1753-6405.2009.00364.x 19413861

[B48] Entman RM Framing: toward clarification of a fractured paradigm J Commun 1993 43 51 58 10.1111/j.1460-2466.1993.tb01304.x

[B49] Kitzinger J Devereux E Framing and frame analysis Media studies: key issues and debates 2007 SAGE, London 134 161

[B50] Terkildsen N Schnell FI Ling C Interest groups, the media, and policy debate formation: an analysis of message structure, rhetoric, and source cues Polit Commun 1998 15 1 45 61 10.1080/105846098199127

[B51] Philo G Devereux E News content studies, media group methods and discourse analysis: a comparison of approaches Media studies: key issues and debates 2007 SAGE Publications, London 101 133

[B52] Hoffman LH Slater MD Evaluating public discourse in newspaper opinion articles: Values-framing and integrative complexity in substance and health policy issues J Mass Commun Q 2007 84 1 58 74

[B53] Gross K Framing pesuasive appeals: episodic and thematic framing, emotional response, and policy opinion Political Psychol 2008 29 2 169 192 10.1111/j.1467-9221.2008.00622.x

[B54] Gross K Brewer PR Sore losers: News frames, policy debates, and emotions Harv Int J Press-Polit 2007 12 1 122 133 10.1177/1081180X06297231

[B55] Chapman S McLeod K Wakefield M Holding S Impact of news of celebrity illness on breast cancer screening: Kylie Minogue's breast cancer diagnosis Med J Aust 2005 183 5 247 250 16138798 10.5694/j.1326-5377.2005.tb07029.x

[B56] Casswell S Public Discourse on Alcohol Health Promot Internation 1997 12 3 251 257 10.1093/heapro/12.3.251

[B57] Fogarty AS Chapman S Framing and the marginalisation of evidence in media reportage of policy debate about alcopops, Australia 2008–2009: Implications for advocacy Drug Alcohol Rev 2010 569 576 21355902 10.1111/j.1465-3362.2010.00253.x

[B58] Fogarty A Chapman S Australian television news coverage of alcohol, health and related policies, 2005 to 2010: implications for alcohol policy advocates Aust NZ J Publ Healin press 10.1111/j.1753-6405.2012.00933.x 23216493

[B59] Australian Health News Research Collaboration Television news database 2011 [accessed on 02/02/2011]. Available from: http://sydney.edu.au/medicine/public-health/AHNRC/videos/login

[B60] Neuendorf KA The Content Analysis Guidebook 2002 Sage Publications, Thousand Oaks, California

[B61] Zhongdang P Kosicki GM Framing analysis: an approach to news discourse Polit Commun 1993 10 1 55 75 10.1080/10584609.1993.9962963

[B62] Sim J Wright CC The Kappa statistic in reliability studies: use, interpretation, and sample size requirements Phys Ther 2005 85 3 257 268 15733050

[B63] Fleiss JL Statistical methods for rates and proportions 1973 John Wiley & Sons, Oxford, England

[B64] Commonwealth of Australia Taking preventative action - a response to Australia: the healthiest country by 2020 2010 [accessed on 12/03/2012]. Available from: http://www.preventativehealth.org.au/internet/preventativehealth/publishing.nsf/Content/6B7B17659424FBE5CA25772000095458/$File/tpa.pdf

[B65] National Preventative Health Taskforce Tobacco control in Australia: making smoking history. Technical report 2 2009 Tobacco Working Group, Australian Government, Canberra

[B66] Chapman S Public health advocacy and tobacco control: making smoking history 2007 Malden MA: Blackwell Publishing, Oxford

[B67] Nicholls J Everyday, everywhere: alcohol marketing and social media—Current Trends Alcohol Alcohol 2012 23 2012 10.1093/alcalc/ags043 22532575

[B68] Ridout B Campbell A Ellis L ‘Off your Face(book)’: Alcohol in online social identity construction and its relation to problem drinking in university students. Drug Alcohol Rev 2011 10.1111/j.1465-3362.2010.00277.x 21355935

[B69] Nicholls J UK news reporting of alcohol: an analysis of television and newspaper coverage Drug-Educ Prev Policy 2011 18 3 200 206 10.3109/09687631003796453

[B70] Cancer Council Victoria New national alliance formed to reduce harm from alcohol 2010 [accessed on 26/04/2012]. Available from: http://www.cancervic.org.au/naaa-formed.html

[B71] Alcohol Advertising Review Board 2012 [accessed on 27/04/2012]. Available from: http://www.alcoholadreview.com.au/

[B72] Tobin CL Fitzgerald JL Livingstone C Thomson L Harper TA Support for breaking the nexus between alcohol and community sports settings: Findings from the VicHealth community attitudes survey in Australia Drug Alcohol Rev 2012 10.1111/j.1465-3362.2011.00388.x 22145930

